# Complementary Treatment of Breast Cancer Cells with Different Metastatic Potential with Iscador Qu in the Presence of Clinically Approved Anticancer Drugs

**DOI:** 10.3390/cimb46110740

**Published:** 2024-11-05

**Authors:** Ivan Iliev, Iana Tsoneva, Aleksandrina Nesheva, Galya Staneva, Bozhil Robev, Albena Momchilova, Biliana Nikolova

**Affiliations:** 1 Institute of Experimental Morphology, Pathology and Anthropology with Museum, Bulgarian Academy of Sciences, Acad. G. Bonchev Str., Bl. 25, 1113 Sofia, Bulgaria; taparsky@abv.bg; 2 Institute of Biophysics and Biomedical Engineering, Bulgarian Academy of Sciences, Acad. G. Bonchev Str., Bl. 21, 1113 Sofia, Bulgaria; itsoneva@bio21.bas.bg (I.T.); nescheva@gmail.com (A.N.); albena_momchilova@abv.bg (A.M.); 3 Department of Medical Oncology, University Hospital “Sv. Ivan Rilski”, 15 Acad. Ivan Geshov Blvd, 1431 Sofia, Bulgaria; bostro@abv.bg

**Keywords:** Iscador Qu, antiproliferative activity, cytotoxicity, breast cancer cells, anticancer drugs, complementary treatment

## Abstract

European mistletoe extract (Iscador Qu) has been studied for decades, but it has not ceased to arouse scientific interest. The purpose was to investigate the impact of Iscador Qu on the antiproliferative potential of 11 standard chemotherapeutic agents on two breast cancer cell lines: MCF-7 low-metastatic and MDA-MB-231 high-metastatic and control cell lines (MCF-10A). MTT-dye reduction assay, FACS analysis, and PI staining were utilized. The most promising combinations acting against the MDA-MB-231 cell line were observed upon the simultaneous application of Iscador Qu (80 µg/mL) and Docetaxel, with 4-fold reduction in IC_50_. An antagonistic effect was found under treatment with Cisplatin and Iscador Qu (1.5-fold increase in IC_50_). The response of the low-metastatic breast cancer cell line MCF-7 to the tested combinations was different compared to the high-metastatic one. The most pronounced cytotoxic effect was found for the combination of Oxaliplatin and Iscador Qu (20 µg/mL) (5.2-fold IC_50_ reduction). An antagonistic effect for MCF-7 line was also observed when combinations with Olaparib and Tamoxifen were applied. This in vitro study offers new combinations between Iscador Qu and standard chemotherapeutic agents that hold great promise in establishing breast cancer therapeutic protocols compared to traditional monotherapies.

## 1. Introduction

Breast cancer is the most common type of malignancy among women all over the world. The tumor malignances differ by molecular characteristics, prognosis, and invasiveness, as breast cancer progression varies significantly in different cases. Many treatment methods are currently established, including surgery, radiation therapy, biological therapy, hormone therapy, and chemotherapy. Despite all the above methods, recurrence is still a problem in breast cancer patients. Possibly due to side effects, drug toxicity in normal cells, and the aggressive behavior of some types of tumors, the obtained results are still unsatisfactory. These therapeutic results motivate the search for new approaches or combinations of already established therapeutic methods. The combination of drugs is a commonly used approach in the treatment of complex diseases [1]. Mainly, efforts are directed toward achieving lower toxicity, avoiding drug resistance, and attacking multiple targets. A promising combination that can be used includes a medicinal plant with a conventional cytostatic, an approach that can reduce the dose of the drugs administered, thus decreasing the side effects.

*Viscum album* (European mistletoe) is a hemiparasitic plant growing on the branches of different trees. Mistletoe obtains from the host water and mineral or organic components. The drug Iscador Qu, comprising extracts from European mistletoe (*Viscum album*), has been used in alternative or supplementary medicine. The leaves of mistletoe are evergreen and do not fall off in winter. European mistletoe has a specific life cycle and extraordinary molecular and biochemical properties. The absence of Complex I in the mitochondria in *Viscum album* leads to reorganization of the respiratory chain [2]. Huge differences have also been registered in the genome of *V. album*; i.e., the largest genome of any flowering plant was found in the seeds of *V. album* [3]. Schroder et al. identified nearly 200 new mitochondrial proteins; four of them are connected to Complex I, whereas all of them have a secondary function not related to respiratory electron transport [4]. The anticancer activities of mistletoe are probably due to the low-molecular-mass proteins, namely lectins and viscotoxins [5,6,7]. Other compounds in *Viscum album* extracts with particular biochemical importance are phenolic acids, phenylpropanoids, flavonoids, triterpenes, and phytosterols [8].

Regardless of the fact that the European mistletoe has been studied for decades, and its products have been applied in medicine, it has not ceased to arouse scientific interest. It has been shown to be cytotoxic to different types of cells, including animal, bacterial, and fungal cells [9,10]. In vitro studies on the simultaneous application of certain combinations of cytostatic drugs with Iscador Qu on human cancer cells were previously published by Weissenstein et al. [11].

First-line breast cancer treatment depends on several factors: the type of breast cancer, its stage, and the patient’s overall health. Chemotherapy is most effective when more than one drug is used at the same time. A combination of two or three drugs is usually given. Common cytostatic drugs include anthracyclines such as doxorubicin and epirubicin, taxanes such as paclitaxel and docetaxel, 5-fluorouracil (5-FU), or carboplatin. The aim of this study was to investigate the potential of Iscador Qu to change the antiproliferative and cytotoxic in vitro effectiveness of 11 standard chemotherapeutic agents with different origins, molecular structures, weight, and hydrophilicity on two breast cancer cell lines with different metastatic potentials (MCF-7 as the low-metastatic and MDA-MB-231 as the high-metastatic line) and a control cell line (MCF-10A).

## 2. Materials and Methods

### 2.1. Anticancer Drugs

**Scheme 1:**
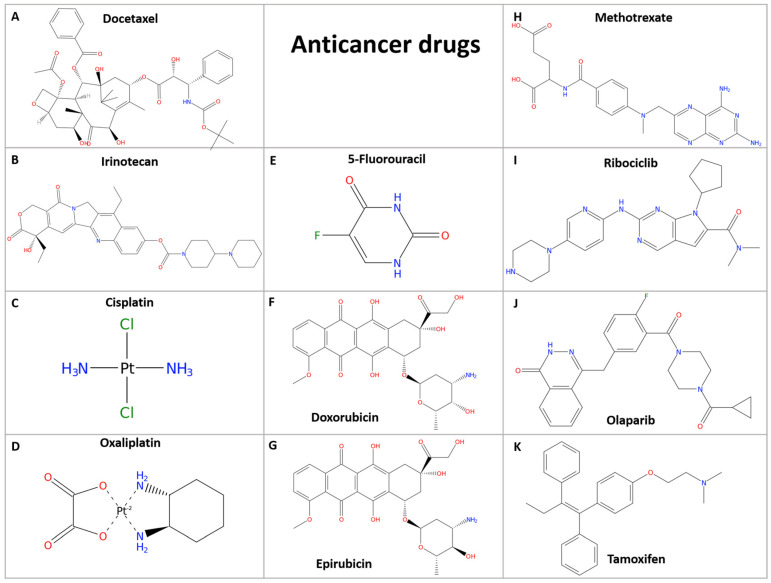
Chemical structure of all studied 11 anticancer drugs.

The anticancer drugs were purchased from the following brands: Docetaxel (Novamed Trading EOOD, Sofia, Bulgaria); Irinotecan, Cisplatin, Oxaliplatin, and Doxorubicin (Accord Healthcare Ltd., London, U.K.); 5-Fluorouracil, Epirubicin, and Methotrexate (Ebewe Pharma GmbH, Unterach, Austria); Ribociclib (Novartis Europharm Ltd., Dublin, Ireland); Olaparib (Astra Zeneca, Cambridge, U.K.); and Tamoxifen (Sopharma AD, Sofia, Bulgaria). The chemical structures of the anticancer drugs are shown in Scheme 1. A short introduction of the drug as molecular weight and chemical formula are presented as well as their main antiproliferative mechanisms.

Docetaxel: MW: 807.8792 g·mol^−1^; chemical formula: C_43_H_53_NO_14_; photosensitive, complex diterpenoid, semisynthetic analogue of paclitaxel [12]. Docetaxel interferes with microtubules implicated in chromosomes moving during mitosis (cell division). Docetaxel blocks cell division and stops cell division and growth.

Irinotecan: MW: 623.14 g·mol^−1^; chemical formula: C_33_H_38_N_4_O_6_. Irinotecan is an antineoplastic enzyme inhibitor primarily used in the treatment of colorectal cancer [13].

Cisplatin: MW: 300.05 g·mol^−1^; chemical formula: Cl_2_H_6_N_2_Pt. Cisplatinum or cis-diamminedichloroplatinum (II) (CDDP) is a platinum coordination complex with potent antineoplastic activity inducing apoptosis in cancer cells, possibly by caspase-3 activation [14]. 

Oxaliplatin: MW: 397.29 g·mol^−1^; chemical formula: C_8_H_14_N_2_O_4_Pt. Oxaliplatin is composed of Cisplatin, as its two amine groups are replaced by diamino cyclohexane (DACH) groups to provide a stronger antitumor effect by forming adducts with DNA [15].

5-Fluorouracil: MW: 130.08 g·mol^−1^; chemical formula: C_4_H_3_FN_2_O_2_. 5-Fluorouracil is a pyrimidine analog that inhibits the thymidylate synthase related to DNA replication.

Doxorubicin: MW: 543.5193 g·mol^−1^; chemical formula: C_27_H_29_NO_11_. It is an anthracycline antibiotic that inhibits topoisomerase II. Doxorubicin binds to nucleic acids, thereby impeding nucleic acid synthesis.

Epirubicin: MW: 543.5 g·mol^−1^; chemical formula: C_27_H_29_NO_11_. It is a 4′-epimer of doxorubicin and also inhibits topoisomerase II and DNA helicase activity. Epirubicin yields less myelosuppression and non-hematologic and cardiac toxicities compared to Doxorubicin.

Methotrexate: MW: 454.44 g·mol^−1^; chemical formula: C_20_H_22_N_8_O_5_. Methotrexate indirectly inhibits cell division via blocking the folate-related enzymes catalyzing the conversion of dihydrofolate to tetrahydrofolate.

Ribociclib: MW: 434.548 g·mol^−1^; chemical formula: C_23_H_30_N_8_O. Ribociclib acts via inhibiting two cyclin-dependent kinase 4 and 6 (CDK4/6), thus decreasing cell growth and division.

Olaparib: MW: 435.08 g·mol^−1^; chemical formula: C_24_H_23_FN_4_O_3_. Olaparib acts via inhibiting PARP, which is implicated in cellular repairing.

Tamoxifen: MW: 371.515 g·mol^−1^; chemical formula: C_26_H_29_NO. Tamoxifen is a selective estrogen response modifier, protein kinase C inhibitor, and anti-angiogenetic factor.

Iscador Qu: Extract from EU *Viscum album*. Iscador Qu 20 mg/mL injection solution (Iscador AG, Lörrach, Germany) was used.

### 2.2. Cell Lines

Breast cell lines (MCF-10A, MCF-7, and MDA-MB-231) were purchased from the American Type Culture Collection—ATCC (Manassas, VA, USA). MCF-7 and MDA-MB-231 were cultivated in Dulbecco’s modified Eagle’s medium (DMEM), supplemented with 10% fetal bovine serum (FBS), 1% sodium pyruvate, and 1% MEM non-essential amino acids (NEAA) without antibiotics. The non-tumorigenic cell line (MCF-10A) was cultivated in DMEM medium (Sigma-Aldrich, St. Louis, MO, USA) and supplemented with 10% FBS, 1% sodium pyruvate, 1% non-essential amino acids (NEAA), 20 ng/mL human epidermal growth factor (hEGF), 10 μg/mL insulin, and 0.5 μg/mL hydrocortisone without antibiotics. All cell lines were maintained in a humidified atmosphere with 5% CO_2_ at 37 °C.

### 2.3. In Vitro Antiproliferative Activity

The antiproliferative activity was tested via using the standard MTT-dye reduction assay described by Mosmann [16]. The method is based on the metabolism of the tetrazolium salt MTT to insoluble formazan. The formazan absorption was registered using a microplate reader at λ = 540 nm. The measured absorption is an indicator of cell viability and metabolic activity. Antiproliferative activities are expressed as IC_50_ values (concentrations required for 50% inhibition of cell growth) and were calculated using non-linear regression analysis (GraphPad Prism 8.0.2 Software, San Diego, CA, USA).

### 2.4. FACS Analysis

MCF10A, MCF-7 and MDA-MB231 cells (7 × 10^6^ cells/well) were seeded in 14-well plates. After 24 h incubation, they were treated with Iscador and were incubated for another 24 h in full culture media. Non-adherent cells were washed with PBS pH 7.4. Following the incubation, the cells were detached through trypsinization and were labeled with an Anexin V-FITC apoptosis detection kit (Sigma-Aldrich, St. Luis, MO, USA) according to the manufacturer’s instructions [17]. The analysis was performed with BD FACS DIVA software (BD Biosciences, Franklin Lakes, NJ, USA).

### 2.5. Cell Cycle Analysis by Propidium Iodide (PI) Staining

After trypsinization, the cells (MCF7, MDA-MB-231, and MCF10A) were resuspended in medium + 10% FCS and centrifuged (1000 rpm, 5 min); then, the pellet was suspended in PBS (1 mL) and fixed with EtOH. After suspension in 2.5 mL pre-cold 70% EtOH, the cells were incubated on ice for 15 min. The staining procedure was as follows: The pellet was suspended in a 500 μL PI solution in PBS (final concentration of PI 1 μg/mL) and 0.1 mg/mL RNAse A, and 0.05% Triton X-100 was added. Following the incubation for 10 min at 37 °C, the cells were transferred to the flow cytometer, and cell fluorescence was measured [18]. The samples were measured by flow cytometry (LSR II, BD Biosciences, Franklin Lakes, NJ, USA) and analyzed using BD FACS DIVA software v. 6.1.3 (BD Biosciences). The control samples of all cell lines underwent the same treatment but without the adding any anticancer drugs or Iscador Qu.

### 2.6. Statistical Analysis

The statistical analysis included application of one-way ANOVA followed by Bonferroni’s post hoc test. *p* < 0.05 was accepted as the lowest level of statistical significance. All results are presented as mean ± SD. All experiments were performed in triplicate.

## 3. Results and Discussion

The breast cell line MDA-MB-231 is a high-metastatic, triple-negative line considered as the most dangerous type of breast cancer, and it is commonly used to model the late stage of the disease. MCF-7 is a less invasive, hormone-dependent, luminal-type breast cancer cell line, whereas the MCF-10A line is the non-malignant one. In our recently published study [19], we investigated the effect of Iscador Qu and M on the phototoxicity, cytotoxicity, antiproliferative activity, actin cytoskeleton organization and migration, changes in ξ-potential of cells, and membrane lipid order on these three breast cell lines with different metastatic potential: MCF-10A (control), MCF-7 (low-metastatic), and MDA-MB-231 (high-metastatic) cells. We also found that the antiproliferative effect of Iscador species were dose-dependent and related to the metastatic potential of the studied cell lines. We further established that Iscador Qu demonstrated higher selectivity for both cancer cell lines compared to Iscador M. We determined the IC_50_ value of each of the studied cell line as follows: 311 μg/mL for MCF-10A, 40 μg/mL for MCF-7, and 92 μg/mL for MDA-MB-231.

Based on these results, we designed the present research with the aim to analyze the effect of the combined treatment of breast cancer cells with Iscador Qu and several conventional cytostatic drugs. For each cell line, we chose to investigate two concentrations of Iscador Qu close to IC_50_ and IC_10_. The lower one was selected so that there was no significant difference in cell viability compared to the control. Thus, to form the combinations with the cytostatic dugs, we selected the following concentrations of Iscador Qu: 80 and 40 μg/mL for MDA-MB-231, 20 and 10 μg/mL for MCF-7, and 100 and 50 μg/mL for MCF-10A. The figures below present the antiproliferative activity of 11 clinically proven cytostatics applied concurrently with Iscador Qu on each of the three tested cell lines.

### 3.1. Docetaxel

Docetaxel is a clinically well-established antimitotic chemotherapy medication used for treatment of different types of cancer, including breast cancer [20]. Docetaxel is a complex diterpenoid molecule and a semisynthetic analogue of paclitaxel. Docetaxel reversibly binds to the microtubule with high affinity in a 1:1 stoichiometric ratio, allowing it to prevent cell division and promote to cell death. Compared to Paclitaxel, Docetaxel is 2-fold more potent as an inhibitor of microtubule depolymerization. Docetaxel binds to microtubules but does not interact with dimeric tubulin [12]. Docetaxel interferes with the normal function of microtubule growth by arresting their activity by hyper-stabilizing their structure. This destroys the cell’s ability to use its cytoskeleton in a flexible manner. Specifically, Docetaxel binds to the β-subunit of tubulin.

**Figure 1:**
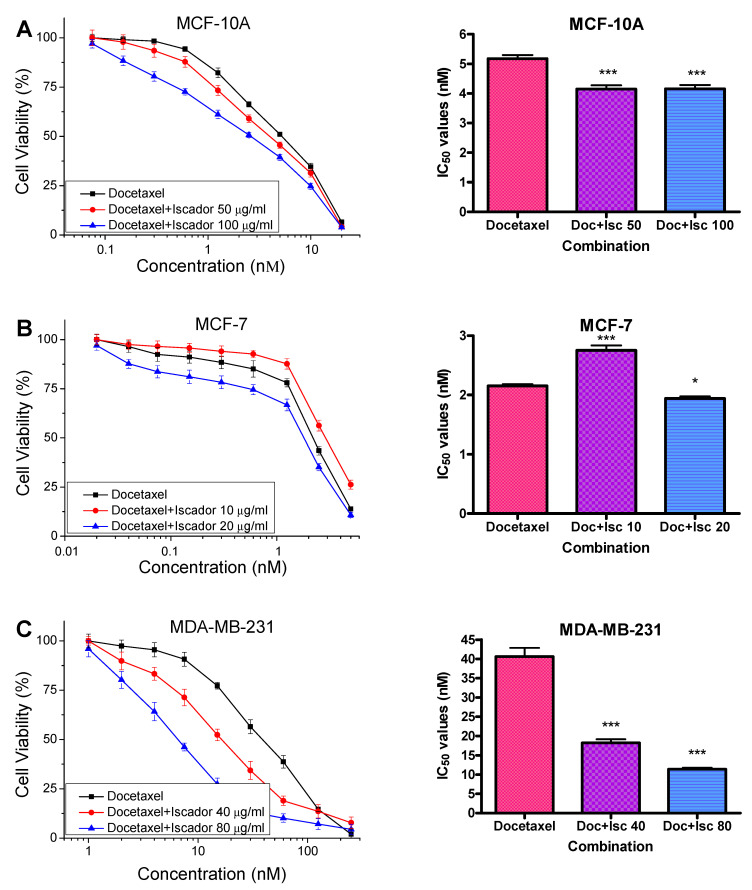
Antiproliferative activity of Docetaxel in combination with two concentrations of Iscador Qu against (**A**) MCF-10A, (**B**) MCF-7, and (**C**) MDA-MB-231. Mean IC_50_ ± SD [nM] from (**A**–**C**). *** *p* < 0.001 and * *p* < 0.05; n = 6.

Antiproliferative activity of Docetaxel in combination with two concentrations of Iscador Qu on three breast lines is presented in Figure 1. The highest effect of the combined treatment was achieved for the aggressive metastatic cell line MDA-MB-231. We found a more than 2-fold decrease in the IC_50_ value in combination of Docetaxel with 40 µg/mL Iscador Qu and an almost 4-fold decrease in the combination with 80 µg/mL Iscador Qu. The IC_50_ was slightly reduced in the case of normal non-cancer cell line MCF-10A in the presence of Docetaxel with both tested concentrations of Iscador Qu (50 or 100 µg/mL). The IC_50_ for the combined treatment was about 4.2 nM for both concentrations of Iscador Qu, whereas single treatment with Docetaxel exhibited 5.18 nM. The combined treatment of the control MCF-10A line gave only a 1.2-fold IC_50_ decrease. Surprisingly, the combined treatment on the low-metastatic cell line MCF-7 was able to induce IC_50_ increase at the low concentration of Iscador Qu and a very slight IC_50_ decrease at the higher concentration.

### 3.2. Irinotecan

**Figure 2:**
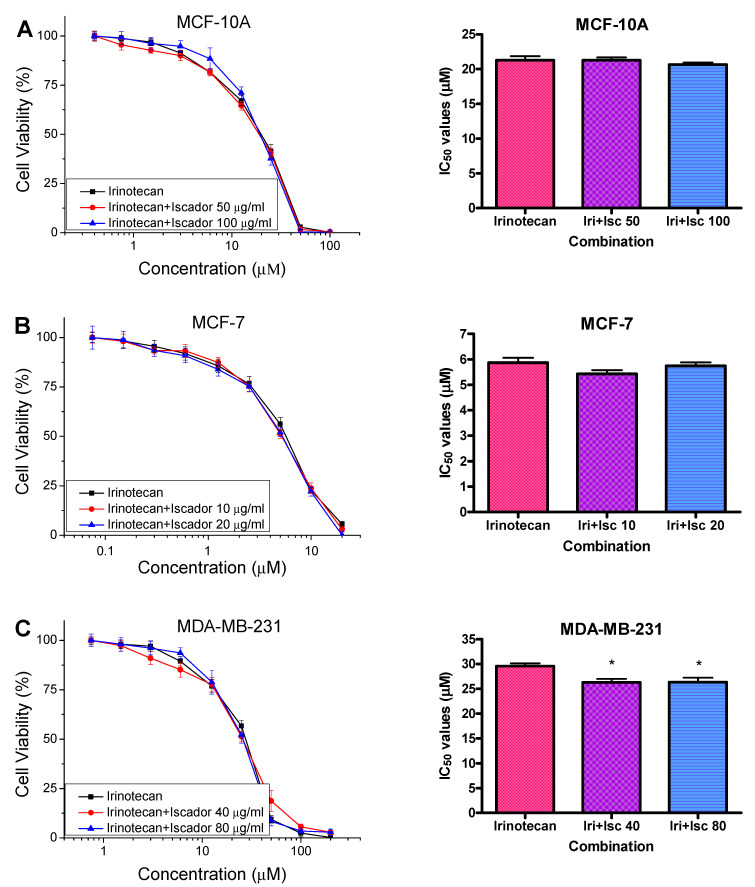
Antiproliferative activity of Irinotecan in combination with two concentrations of Iscador Qu against (**A**) MCF-10A, (**B**) MCF-7, and (**C**) MDA-MB-231. Mean IC_50_ ± SD [μM] from (**A**–**C**). * *p* < 0.05; n = 6.

Irinotecan is water-soluble derivative from the Chinese tree (*Camptotheca acuminata*). This anticancer drug is a DNA topoisomerase I inhibitor leading to G2/M phase arrest and apoptosis. Furthermore, it was recently shown that topoisomerase I may not be the only target of Irinotecan [21]. The drug is predominately used for colorectal cancer cells [13,21]. The antiproliferative activity of Irinotecan in combination with two concentrations of Iscador Qu on three breast lines is presented in Figure 2.

The results presented in Figure 2 imply that the combined treatment of breast cancer cell lines with Irinotecan and different concentrations of Iscador Qu did not affect IC_50_ for the control MCF-10A and low-metastatic MCF-7 cell lines, whereas a very slight IC_50_ decrease was observed for the MDA-MB-231 line. Both Irinotecan and its active metabolite SN38, which exhibits 100- to 1000-fold greater anticancer activity, act predominantly by inducing apoptosis. Lately, its additional antiproliferative mechanism was revealed. By using the electroporation-induced internalization of SN38/Irinotecan in the cells, it was detected that ferroptosis, a type of cancer cell death related to ROS generation, is produced via iron-catalyzed Fenton’s reactions [22]. As already mentioned, Irinotecan and its derivatives are able to induce apoptosis or ferroptosis of colon cancers or of colon cancer cells and are regularly used as drugs against neoplastic colon cancer cells. Topoisomerase inhibitors are not routinely used to treat breast cancer, but the deficiencies in DNA damage repair are typical for the triple-negative breast cancer cells, which lack all three breast cancer-associated proteins (receptors for estrogen, progesterone, and human epidermal growth factor 2).

### 3.3. Cisplatin

**Figure 3:**
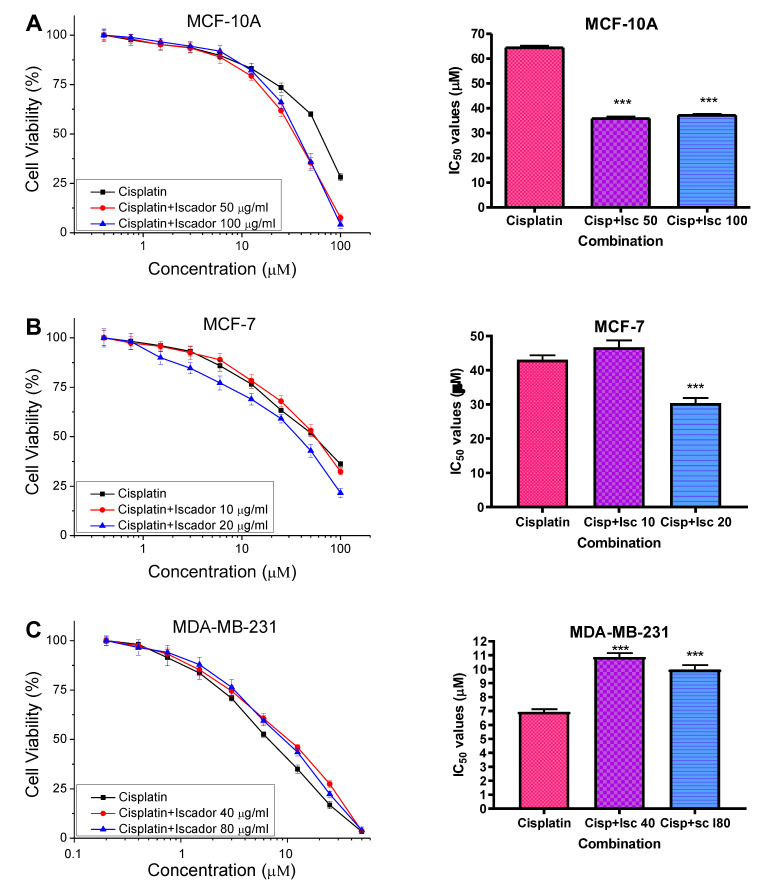
Antiproliferative activity of Cisplatin in combination with two concentrations of Iscador Qu against (**A**) MCF-10A, (**B**) MCF-7, and (**C**) MDA-MB-231. Mean IC_50_ ± SD [μM] from (**A**–**C**). *** *p* < 0.001; n = 6.

The mechanism of action of Cisplatin has been associated with ability to crosslink with the purine bases of DNA, preventing DNA repair and leading to its damage, subsequently inducing cancer cells apoptosis [23]. The antiproliferative activity of Cisplatin in combination with two concentrations of Iscador Qu on three studied breast lines is shown in Figure 3.

The combined treatment with Cisplatin and Iscador Qu enhanced the antiproliferative effect of Cisplatin on the normal cell line MCF-10A, regardless of the applied concentration of Iscador Qu (50 or 100 µg/mL). A similar but less pronounced trend was observed for the low-metastatic MCF-7 line at the higher Iscador Qu concentration. Surprisingly, the combined treatment of the high-metastatic MDA-MB-231 cell line reduced the antiproliferative effect by 1.6-fold.

### 3.4. Oxaliplatin

**Figure 4:**
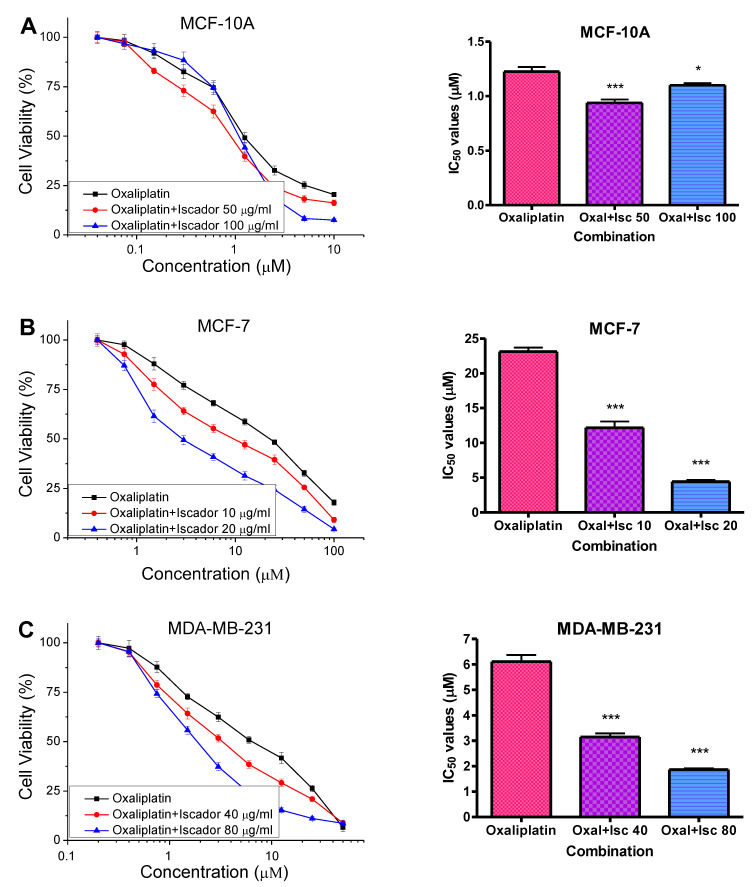
Antiproliferative activity of Oxaliplatin in combination with two concentrations of Iscador Qu against (**A**) MCF-10A, (**B**) MCF-7, and (**C**) MDA-MB-231. Mean IC_50_ ± SD [µM] from (**A**–**C**). *** *p* < 0.001 and * *p* < 0.05; n = 6.

Oxaliplatin is a part of the platinum-based group of anticancer drugs including Cisplatin and Carboplatin. Oxaliplatin was patented in 1976 but approved for medical use in 1996. Oxaliplatin and Cisplatin block the duplication of DNA in cells. At high concentrations, Oxaliplatin suppresses cellular RNA and protein synthesis. The drug induces cell cycle arrest and moderate apoptosis [24]. Ongoing trials have also shown promising results for Oxaliplatin use in non-Hodgkin lymphoma, breast cancer, mesothelioma, and non-small-cell lung cancer [15]. Figure 4 represents the antiproliferative activity of Oxaliplatin in combination with two concentrations of Iscador Qu on the three studied breast lines.

The combined treatment of Oxaliplatin with Iscador Qu increased the antiproliferative effect on all three studied breast cell lines, and this increase was higher in the cancer cell lines. As can be seen from Figure 4, the combination of Oxaliplatin with 10 µg/mL of Iscador Qu led to an almost 2-fold decrease in IC_50_ in the low-metastatic cell line MCF-7 and a 4.5-fold decrease at 20 µg/mL Iscador Qu. A similar trend was observed for the triple-negative high-metastatic cell line MDA-MB-231 with 1.9-fold and 3.3-fold IC_50_ decreases at 40 and 80 µg/mL Iscador Qu, respectively.

### 3.5. 5-Fluorouracil

**Figure 5:**
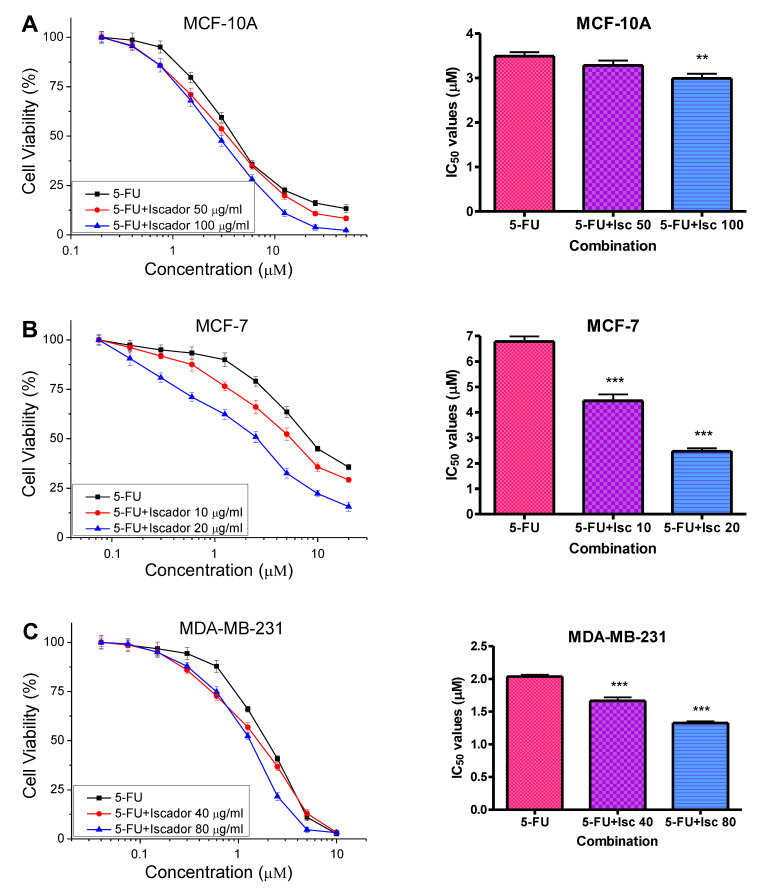
Antiproliferative activity of 5-Fluorouracyl in combination with two concentrations of Iscador Qu against (**A**) MCF-10A, (**B**) MCF-7, and (**C**) MDA-MB-231. Mean IC_50_ ± SD [µM] from (**A**–**C**). *** *p* < 0.001 and ** *p* < 0.01; n = 6.

5-Fluorouracil (5-FU) is among the most frequently administrated chemotherapeutic agents for a wide variety of neoplasms. 5-FU for intravenous injection has been used for treatment of colorectal, esophageal, stomach, pancreatic, and breast cancer. The drug inhibits DNA synthesis by blocking the incorporation of the thymidine nucleotide into the DNA strand [25]. The antiproliferative activity of 5-FU in combination with two concentrations of Iscador Qu on three studied breast lines is shown in Figure 5. This anticancer drug did not change the antiproliferative properties of MCF-10A at 50 µg/mL Iscador Qu but slightly decreased it at 100 µg/mL Iscador Qu. The combined treatment on the low-metastatic breast cancer cell line MCF-7 led to 1.5- and 2.7-fold decreases in IC_50_ at 10 and 20 µg/mL Iscador Qu, respectively.

In the case of the MDA-MB-231 line, the combined treatment induced lower antiproliferative activity compared to MCF-7, as the decrease in IC_50_ varied from 1.2- to 1.5-fold at 40 and 80 µg/mL Iscador Qu, respectively.

### 3.6. Doxorubicin

**Figure 6:**
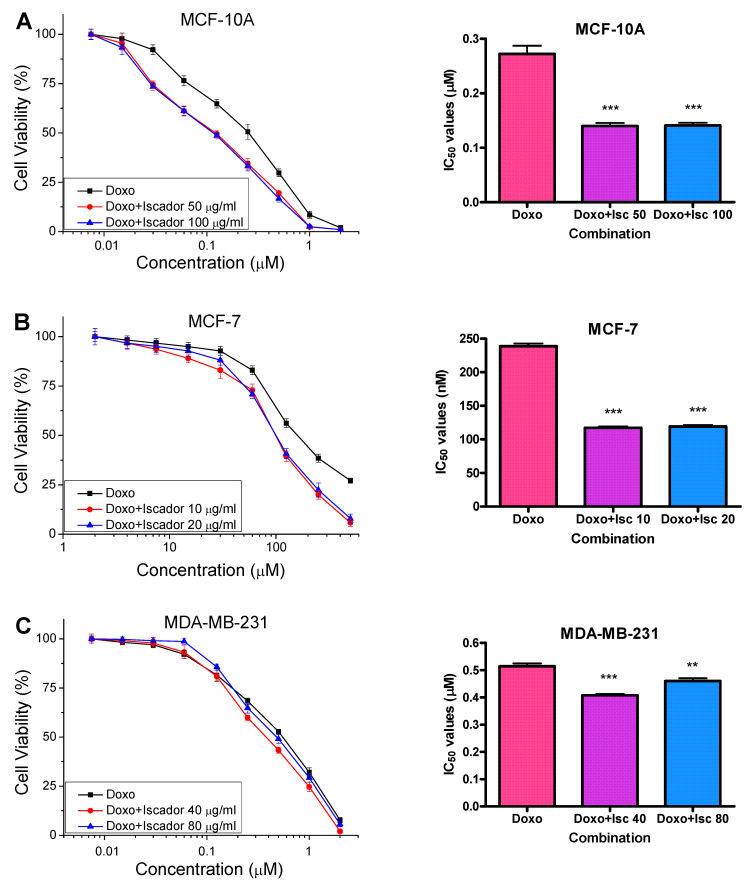
Antiproliferative activity of Doxorubicin in combination with two concentrations of Iscador Qu against (**A**) MCF-10A, (**B**) MCF-7, and (**C**) MDA-MB-231. Mean IC_50_ ± SD from (**A**–**C**). *** *p* < 0.001 and ** *p* < 0.01; n = 6.

The viability of both the MCF-10A and MCF-7 cell lines was about 2-fold reduced under combined treatment of Doxorubicin with both studied concentrations of Iscador Qu (Figure 6A,B). Higher IC_50_ values were found for MDA-MB-231 under combined treatment, which means a lower efficacy of this treatment for the high-metastatic cell line (Figure 6C).

### 3.7. Epirubicin

Epirubicin is the epimer of doxorubicin, with an inversion of the 4′-hydroxyl group on the sugar moiety. This drug is an anthracycline with maximal cytotoxic effects in the S and G2 phases. In vitro studies have been demonstrated that Epirubicin possesses cytotoxicity at least equivalent to that of doxorubicin against a variety of cancer cell lines but exhibits a different toxicity profile, particularly in regard to myelotoxicity and cardiotoxicity [26].

**Figure 7:**
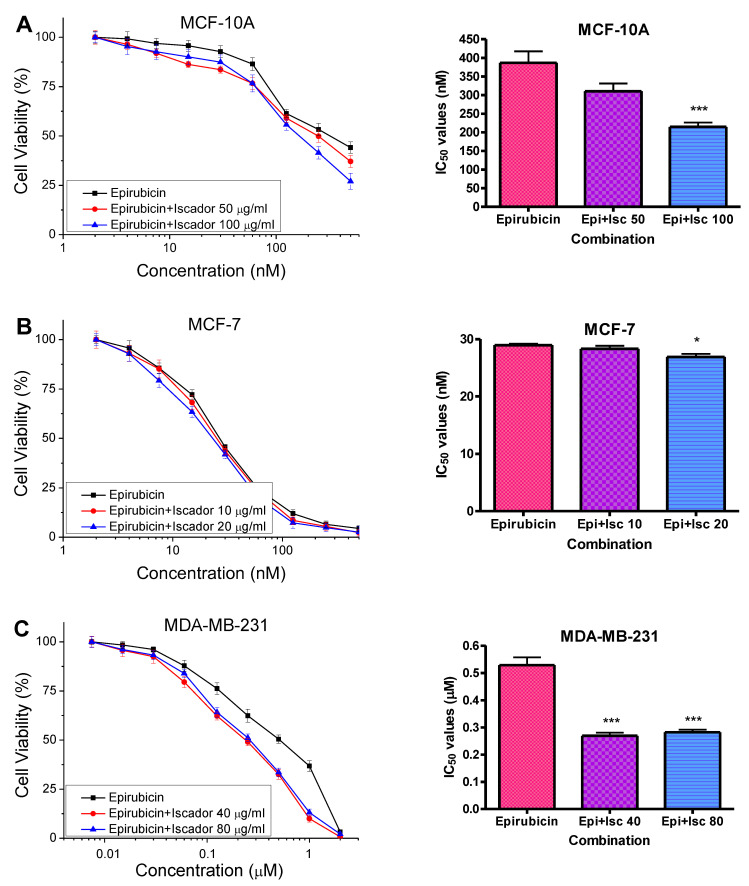
Antiproliferative activity of Epirubicin in combination with two concentrations of Iscador Qu against (**A**) MCF-10A, (**B**) MCF-7, and (**C**) MDA-MB-231. Mean IC_50_ ± SD from (**A**–**C**). *** *p* < 0.001 and * *p* < 0.05; n = 6.

The antiproliferative activity of Epirubicin in combination with two concentrations of Iscador Qu on three studied breast lines is shown in Figure 7. A high dose of Epirubicin (386.9 µM) was required to achieve IC_50_ for the non-tumor cell line MCF10-A (Figure 7A). Under combined treatment, a 2-fold decrease in IC_50_ was found for 100 μg/mL Iscador Qu (Figure 7A). The hormone-sensitive MCF-7 cell line did not exhibit significant sensitivity in the antiproliferative activity to the combined treatment. In contrast, the combined treatment on triple-negative highly metastatic cell line MDA-MB-231 showed a 2-fold decrease in IC_50_ at both concentrations of Iscador Qu (40 and 80 µg/mL) (Figure 7C).

### 3.8. Methotrexate

Methotrexate (MTX) is a drug used for treatment of rheumatoid arthritis (RA) and other forms of inflammatory arthritis. Understanding the mechanism of action of Methotrexate could be instructive in the appropriate use of the drug and in the design of new regimens for the treatment of autoimmune diseases. Although Methotrexate is one of the first examples of intelligent drug design, with multiple mechanisms potentially contributing to the anti-inflammatory actions of methotrexate, including the inhibition of purine and pyrimidine synthesis, transmethylation reactions, translocation of nuclear factor-κB (NF-κB) to the nucleus, signaling via the Janus kinase (JAK)–signal transducer and activator of transcription (STAT) pathway, and nitric oxide production [27].

**Figure 8:**
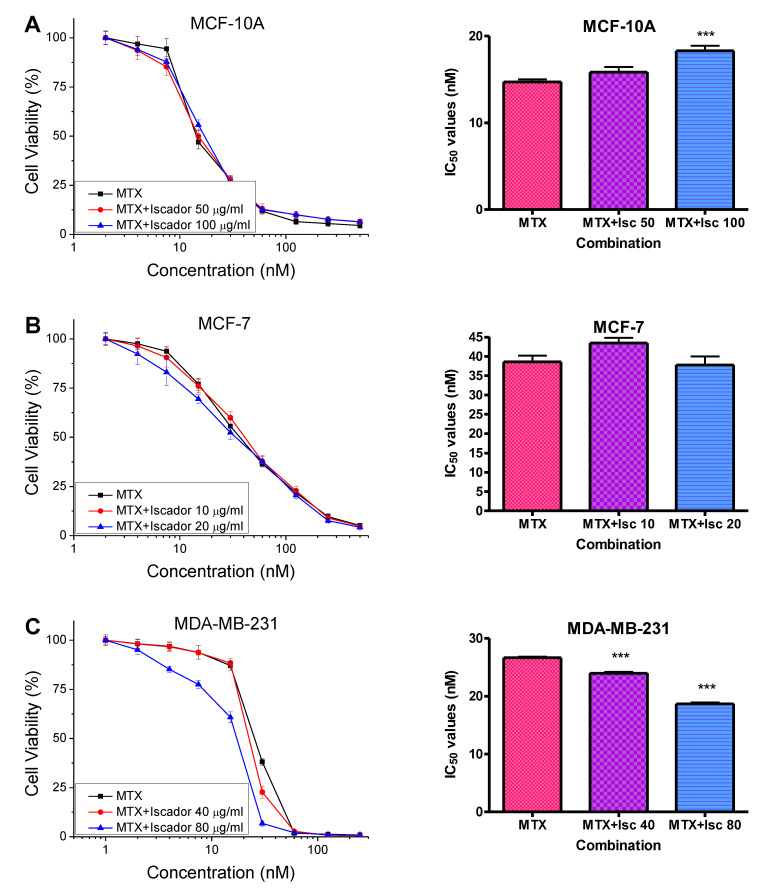
Antiproliferative activity of Methotrexate in combination with two concentrations of Iscador Qu against (**A**) MCF-10A, (**B**) MCF-7, and (**C**) MDA-MB-231. Mean IC_50_ ± SD [nM] from (**A**–**C**). *** *p* < 0.001; n = 6.

As can be seen from Figure 8, the combined treatment of MCF-10A with methotrexate and Iscador Qu applied in two concentrations did not reduce the IC_50_ dose at 50 µg/mL Iscador Qu and even slightly increased it at 100 µg/mL. The low-metastatic MCF-7 line did not exhibit any sensitivity to the combined treatment, whereas a 1.5-fold reduction in IC_50_ was documented for the high-metastatic MDA-MB-231 line at 80 µg/mL Iscador Qu.

### 3.9. Olaparib

Olaparib acts by blocking the repair of single-strand DNA. It is recommended for treatment of high-risk and early breast cancer. It is used predominately for ovarian cancer, which is one of the most common cancers in women and has a high mortality rate [28]. For patients with high-risk HER2-negative early breast cancer, the application of Olaparib as an adjuvant after completion of local treatment and neoadjuvant or adjuvant chemotherapy was associated with significantly longer survival compared to placebo. Additionally, Olaparib shows limited side effects on global patient-reported quality of life [29]. Usually, Iscador Qu, similarly to Olaparib, is used alone after chemotherapy as an adjuvant medicament. It is also possible that the simultaneous use of two medications with an adjuvant effect could inhibit the anticancer activity of one another [28,29].

**Figure 9:**
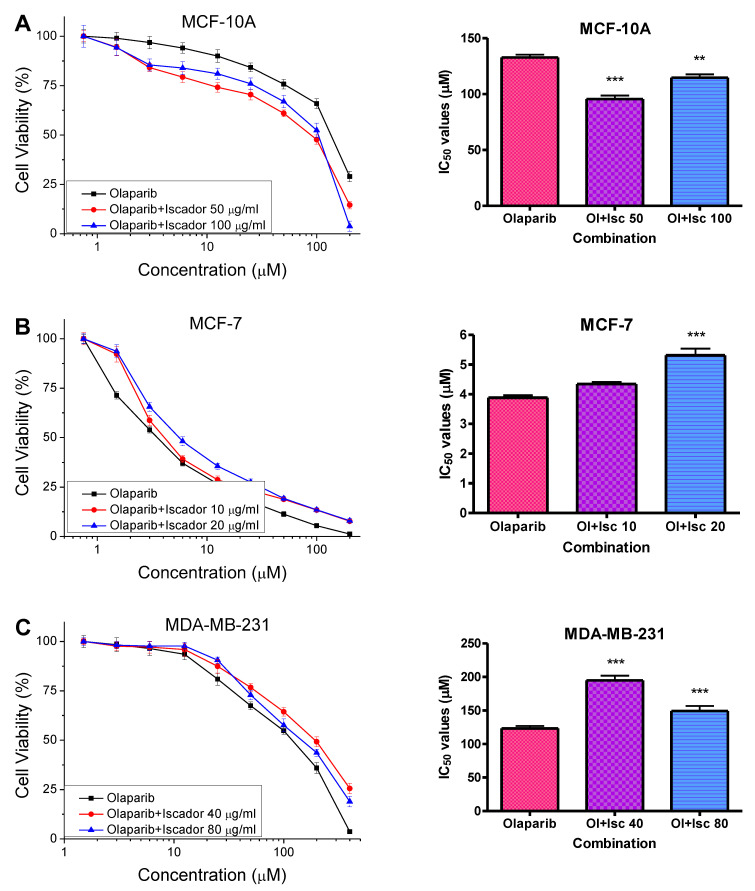
Antiproliferative activity of Olaparib in combination with two concentrations of Iscador Qu against (**A**) MCF-10A, (**B**) MCF-7, and (**C**) MDA-MB-231. Mean IC_50_ ± SD [µM] from (**A**–**C**). *** *p* < 0.001 and ** *p* < 0.01; n = 6.

The antiproliferative activity of Olaparib in combination with two concentrations of Iscador Qu on three studied breast lines is shown in Figure 9. A slight decrease in IC_50_ dose was observed under the combined treatment of the control MCF-10A cell line (Figure 9A). In contrast, an increase in IC_50_ dose was found for both the low- and high-metastatic cell lines in response to the combined treatment. A negative treatment effect was obtained for the MCF-7 cell line at 20 µg/mL Iscador Qu and for the MDA-MB-231 line at 40 µg/mL Iscador Qu.

### 3.10. Ribociclib

Ribociclib is a kinase inhibitor used to treat HR+, HER2-, advanced, or metastatic breast cancer. It is a selective cyclin-dependent kinase inhibitor, a class of drugs that helps slow the progression of cancer by blocking two proteins called cyclin-dependent kinase 4 and 6 [30].

**Figure 10:**
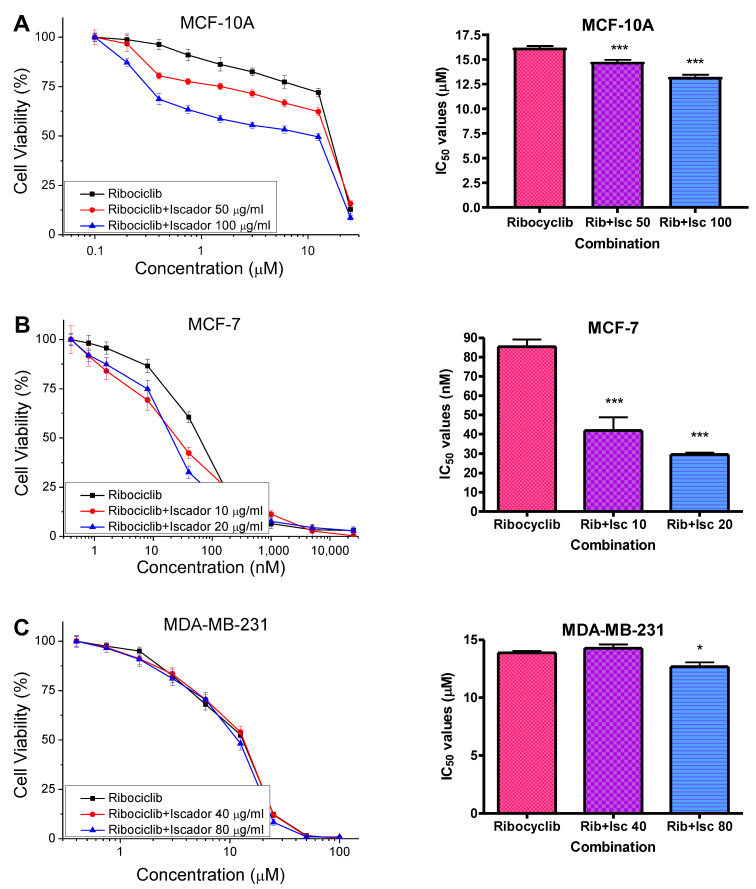
Antiproliferative activity of Ribociclib in combination with two concentrations of Iscador Qu against (**A**) MCF-10A, (**B**) MCF-7, and (**C**) MDA-MB-231. Mean IC_50_ ± SD from (**A**–**C**). *** *p* < 0.001 and * *p* < 0.05; n = 6.

The obtained data for antiproliferative activity under combined treatment of studied cell lines with Ribociclib and Iscador Qu are presented in Figure 10. The results indicate that the combined treatment of the control MCF-10A and the high-metastatic MDA-MB-231 cell lines did not significantly affect the dose required to achieve IC_50_ (Figure 10A,C), while the low-metastatic cell line MCF-7 responded by 3-fold IC_50_ reduction at 20 µg/mL Iscador Qu and a 2-fold reduction at 10 µg/mL (Figure 10B).

### 3.11. Tamoxifen

Tamoxifen is a hormone-related drug for therapy of breast cancer in both women and men. It lowers the risk of early breast cancer coming back (recurring) after surgery or developing in the other breast. Tamoxifen competitively inhibits the binding of estradiol to estrogen receptors and works by decreasing the growth of cancer cells. It can also control advanced breast cancer for some time. Tamoxifen and Raloxifene are the only drugs approved in the USA to help lower the risk of breast cancer. Tamoxifen has been shown to reduce the risk of breast cancer in women with a higher-than-average risk, but these drugs can have their own risks and side effects [31]. Tamoxifen and Olaparib, similar to Iscador Qu, are used more as adjuvant therapies.

**Figure 11:**
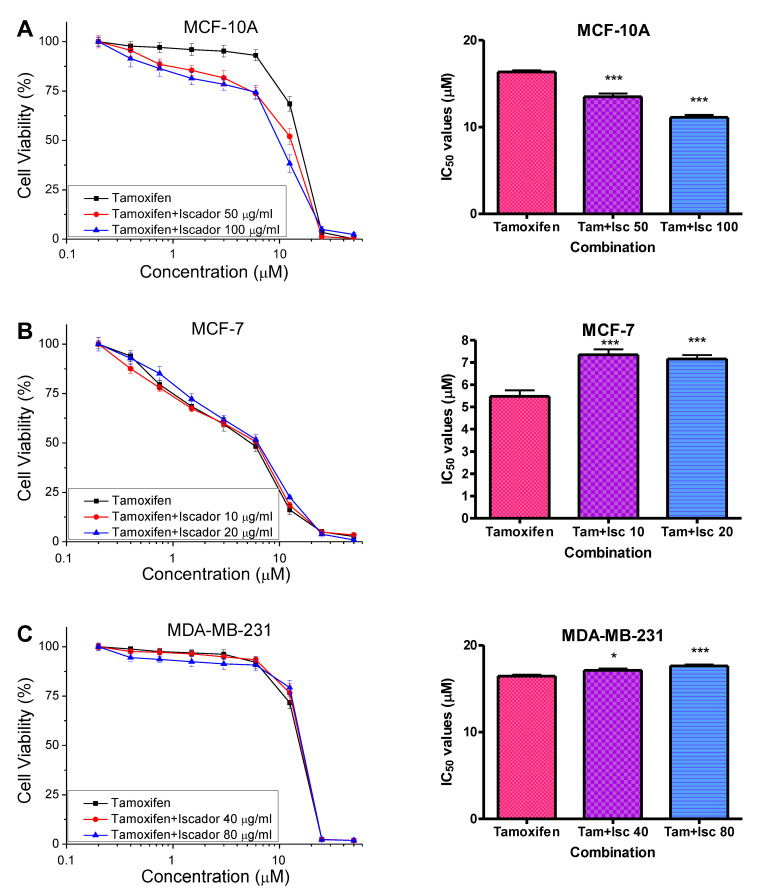
Antiproliferative activity of Tamoxifen in combination with two concentrations of Iscador Qu against (**A**) MCF-10A, (**B**) MCF-7, and (**C**) MDA-MB-231. Mean IC_50_ ± SD [µM] from (**A**–**C**). *** *p* < 0.001 and * *p* < 0.05; n = 6.

The antiproliferative activity of Tamoxifen in combination with two concentrations of Iscador Qu on the three studied breast lines is presented in Figure 11. The data demonstrate that the combined treatment negatively affected the low- and high-metastatic cell lines (Figure 11B,C) compared to the control non-malignant MCF-10A line, where a decrease in IC_50_ dose was observed (Figure 11A). The negative effect under combined treatment was less pronounced in the high-metastatic cell line compared to the low-metastatic one.

The majority of anticancer drugs screened in this study act predominantly on DNA synthesis, repair, and cellular replication. Since all used cytostatic drugs are small in size and have no net charge, it is assumed that they do not interact with the cells by direct electrostatic interactions. They probably internalize the plasma membrane of cells by passive diffusion. The surface electrical charge is specific for the different cell types and represents a critical parameter for neoplastic cells. High-metastatic cancer cells have a higher negative zeta potential due to the negatively charged mobile lactate products of the increased glycolysis, which is like a passport for neoplastic cells and is used in the prognosis and treatment of tumors [32,33]. In our previous study [19], we found that the changes in zeta-potential values of the treated with Iscador Qu cell lines (MCF-10A—control, MCF-7—low-metastatic, and MDA-MB-231—high-metastatic) were in accordance with the established antiproliferative activity rank of the studied cell lines: MCF-7 > MDA-MB-231 > MCF-10A. Furthermore, we established that Iscador Qu was able to change the plasma membrane lipid order of the control MCF-10A and low-metastatic MCF-7 cell lines but not that of the high-metastatic MDA-MB-231 line. Despite that fact, in response to the Iscador Qu treatment, the regular organization of the actin filaments was perturbed in both cancer cell lines compared to the control one. Such changes in the organization of the actin cytoskeleton were shown to inhibit cell adhesions and affect cell survival. On other hand, the wound-healing assay confirmed the decreased motility and ability of the low-metastatic MCF-7 cell line to proliferate but not that of the high-metastatic MDA-MB-231. This was the rationale to screen the combinations of conventional anticancer drugs with Iscador Qu in order to test whether the latter could potentiate drug toxicity at sub-therapeutic concentrations. Table 1 and Table 2 represent the rank of combinations (anticancer drug + Iscador Qu) for every cell line, from the best to the worst combination (having an antagonistic, antiproliferative effect). The rank of combinations is expressed by sorting the relative change of IC_50_ (%) calculated by ((IC_50_ (combination) − IC_50_ (drug)/IC_50_ (drug)). The summarized data were extracted from the original data (Figure 1, Figure 2, Figure 3, Figure 4, Figure 5, Figure 6, Figure 7, Figure 8, Figure 9, Figure 10 and Figure 11).

Based on the data presented in Table 1 and Table 2, the drug combinations were systemized in two groups: (i) the groups more significantly affecting the triple-negative, high-metastatic MDA-MB-231 line (Table 1) and (ii) the groups more significantly affecting the low-metastatic MCF-7 line (Table 1).

As can be seen from Table 1, the most promising combinations acting against MDA-MB-231 are (i) Docetaxel + Iscador Qu 80 µg/mL, with a 72% reduction in IC_50_; (ii) Oxaliplatin + Iscador Qu 80 µg/mL, inducing a 70% reduction in IC_50_; (iii) Epirubicin + Iscador Qu 80 µg/mL, with a 47% IC_50_ reduction; (iv) 5-FU + Iscador Qu 80 µg/mL and Methotrexate + Iscador Qu 80 µg/mL, with about 30% reduction in IC_50_. On the other hand, an antagonistic effect was detected under treatment with Cisplatin + Iscador Qu 80 µg/mL (44% increase in IC_50_) and Olaparib + Iscador Qu 80 µg/mL (22% increase in IC_50_). No significant alteration in IC_50_ was observed using combinations with Doxorubicin, Irinotecan, Ribociclib, and Tamoxifen.

Table 2 presents the relative change of IC_50_ values for the low-metastatic MCF-7 cell line according to the studied combinations. When comparing Table 1 and Table 2, it can be seen that the best combinations for MCF-7 are different compared to those for MDA-MB-231. These differences can be explained by the particular characteristics attributed to low- and high-metastatic cancer cell lines, such as differences in gene expression profiles, lipid and protein metabolism, the shape and dimensions of the cells, cytoskeletal properties, membrane fluidity, and ζ potential, among others. As can be seen from Table 2, the most prominent effect was achieved after the following combined treatment: (i) Oxaliplatin + Iscador Qu 20 µg/mL, with an 81% reduction in IC_50_; (ii) Ribociclib + Iscador Qu 20 µg/mL and 5-FU + Iscador Qu 20 µg/mL, inducing an almost 65% reduction in IC_50_; (iii) Doxorubicin + Iscador Qu 20 µg/mL, with a 50% reduction in IC_50_; (iv) Cisplatin + Iscador Qu 20 µg/mL, with a 30% reduction in IC_50_ and with a 50% reduction in IC_50_. An antagonistic effect was found for the combination of Iscador Qu with Olaparib and Tamoxifen, with 37% and 31% increases in IC_50_, respectively. No effect was observed for combinations with Irinotecan, Methotrexate, and Epirubicin.

It is noteworthy that more combinations exerted a positive effect towards the low-metastatic MCF-7 compared to high-metastatic line, which was not unexpected. Four combinations applied to the MCF-7 cell line exhibited about a 50% reduction in IC_50_ in contrast to MDA-MB-231, where only two combinations satisfied this requirement.

Special attention should be paid to the most effective combinations for both cancer cell lines as well as to the most ineffective ones. The most significant effect was observed for the combination of Oxaliplatin + Iscador Qu 20 µg/mL for the MCF-7 line and Oxaliplatin + Iscador Qu 80 µg/mL for the MDA-MB-231 line, with 81% and 72% reductions in IC_50_, respectively.

In the search of the mechanism of action, the cell cycle arrest induced after treatment with the selected combinations was analyzed. The cell aliquots were collected, and FACS analyses were used to detect the effects of the treatments. A propidium iodide staining assay was performed (Figures S1 and S2).

The solo treatments of cells with Iscador Qu did not affect the cell cycle progression.

Regarding the high-metastatic cell line MDA-MB-231, the treatment with Docetaxel alone induced cell cycle arrest in the G0 phase, while treatment with Cisplatin blocked the cells in the S phase of the cell cycle. This is not surprising and is due to the mechanism of action of the anticancer drugs. Surprisingly, the selected combinations did not alter the effect of the Docetaxel and Cisplatin. As a result of the treatments with both the most effective and the most ineffective combinations, there were no effects different from those of the anticancer drug treatment alone.

The results were similar after treatment of the low-metastatic cell line MCF-7. The effect on the cell cycle arrest of the mono treatment with anticancer drugs Oxaliplatin and Olaparib was arrest in the G2/M phase due to the mechanism of action of drugs and no significant difference after combined treatment with the most effective and the most ineffective ones regarding IC_50_ combinations.

The data above do not show significant and reliable changes using the classical methods for studying the cell cycle changes after combined treatment of cell lines with Iscador and cytostatic drugs. It is well known that the classification of programmed cell death includes 11 mechanisms of cell death, with apoptosis being the most common [34]. Further research is needed in order to elucidate the exact mechanism of cell death, such as autophagy and/or ferroptosis. On the other hand, it is well known that cancer cells have a higher negative membrane charge. The metabolically active cancer cells are known to secrete a large amount of lactate as mobile anions. The increased levels of glucose uptake and lactate secretion could be up to 30 times those of normal cells, which is the basis for cancer-detection technologies such as PET (positron emission tomography) scan diagnostics. Cancer cells may have slightly elevated surface contents of negatively charged immobilized molecules (e.g., sialic acid, phosphatidyl serine, etc.): about 30–50% more than normal cells [35,36,37]. In our previous article [38], we found that the zeta potential of the three investigated lines and in the present study is in the following order: MCF-7 > MDA-MB-231 > MCF-10A, but after treatment with synthetic thienopyrimidines with high antiproliferative activity, the changes in the zeta potential of the three cell lines were not in the same order as in the control studies of the above lines. Henslee et al. [39] showed that the average radii (µm) are MCF-10A—9.25, MCF-7—9.1, and MDA-MB-231—8.93, respectively, with a membrane capacitance (F/m^2^) of MCF-10A—0.0194, MCF-7—0.0186, and MDA-MB-231—0.0163; i.e., the specificity of the electric properties of breast cancer models could be changed under treatment with different drugs. The surface electrical properties also depend on phospholipid metabolism, which should not be overlooked.

Therefore, future research on the role of electrical properties such as ζ potential, electrophoretic mobility, or changes in cell membrane potential would contribute to clarifying the mechanism of action of the combination of Iscador Qu with anticancer drugs.

It is well known from clinical practice that 5-FU more strongly affects slowly dividing cells, which is in good correlation with our data. The IC_50_ value for MCF-7 was 6.787 µM, whereas for MDA-MB-231, it was 2.037 µM. In addition, the combination with Iscador Qu induced a 33% lesser reduction in IC_50_ in MDA-MB-231 compared to the MCF-7 cell line. Our in vitro results are in good accordance with those reported by Pelzer et al. [26,27]. In their clinical trial of treating breast cancer patients with Iscador Qu and 5-FU, the patients had a 5-year follow-up period without recurrence. Furthermore, Weissenstein et al. [11] stated that Iscador Qu at concentrations between 0.1 and 10 μg/mL did not influence the cytotoxic effect of Doxorubicin on two human breast carcinoma cell lines, HCC1937 and HCC1143. At concentrations ≥ 10 μg/mL (up to 100 μg/mL), Iscador Qu led to an additive augmentation of the drug’s cytostatic action. Our results are in very good agreement with those of Weissenstein et al. [7], as we demonstrated that the combined treatment of Doxorubicin + Iscador Qu (10 μg/mL) on the low-metastatic MCF-7 cell line induced a 50% reduction in IC_50_ dose, whereas Doxorubicin + Iscador Qu (40 μg/mL) on the high-metastatic MDA-MB-231 line obtained a 21% reduction in IC_50_ dose.

## 4. Conclusions

A promising concept is the use of combination therapy, where two or more drugs are used to treat the complex causes of the spreading of breast cancer and its division into several subtypes. Combination therapies can have significant advantages over monotherapies, but there are still a lot of challenges to overcome, including drug–drug interaction risks, avoiding drug resistance, decreasing the toxicity, as well as insufficient survival examinations. Furthermore, the combination therapies need to be optimized based on the in vitro and in vivo analyses. In the search for therapies with low doses of cytostatic drugs, low side effects, and negative drug–drug interactions, we tested combinations of 11 anticancer drugs with the natural extract Iscador Qu. Anticancer drugs with similar molecular weight but with different origins and structures were selected. We assessed the antiproliferative activity of the tested combinations towards three epithelial breast cell lines: MCF-10A as a control line and MCF-7 as the low-metastatic and MDA-MB-231 as the high-metastatic lines. Several effective and antagonistic combinations were found for low- and high-metastatic breast cancer cell lines. Even though our study is only in vitro, the results can be useful in designing smart anticancer therapeutic strategies for applying low cytostatic concentrations with maximal preservation of the surrounding non-cancer cells. Additional in vivo studies are needed to evaluate the efficacy of Iscador Qu as an adjuvant alternative anticancer drug with well-pronounced beneficial effects on human well-being.

## Figures and Tables

**Table 1:** Relative change of IC_50_ (%) for the low-metastatic MDA-MB-231 cell line calculated by IC_50 (combination)_ − IC_50 (drug)_/IC_50 (drug)_. The relative change of IC_50_ values is listed from lowest value (the best combination) to highest one (the worst combination, having an antagonistic, antiproliferative effect).

MDA-MB-231 Relative Change of IC_50_ (%)			
Drug	Drug + Iscador Qu 40 µg/mL	Drug	Drug + Iscador Qu 80 µg/mL
Docetaxel	−56.10	Docetaxel	−72.20
Epirubicin	−49.06	Oxaliplatin	−69.56
Oxaliplatin	−48.45	Epirubicin	−47.17
Doxorubicin	−21.15	5-Fluorouracyl	−32.35
5-Fluorouracyl	−18.14	Methotrexate	−29.89
Methotrexate	−11.44	Doxorubicin	−11.54
Irinotecan	−11.13	Irinotecan	−10.99
Ribociclib	2.81	Ribociclib	−8.57
Tamoxifen	4.07	Tamoxifen	7.29
Cisplatin	56.77	Olaparib	21.09
Olaparib	58.15	Cisplatin	43.80

**Table 2:** Relative change of IC_50_ (%) for the low-metastatic MCF-7 cell line calculated by IC_50 (combination)_ − IC_50 (drug)_/IC_50 (drug)_. The relative change of IC_50_ values are listed from lowest value (the best combination) to highest one (the worst combination, having an antagonistic, antiproliferative effect).

MCF-7 Relative Change of IC_50_ (%)			
Drug	Drug + Iscador Qu 10 µg/mL	Drug	Drug + Iscador Qu 20 µg/mL
Ribociclib	−50.59	Oxaliplatin	−80.76
Doxorubicin	−50.00	Ribociclib	−65.88
Oxaliplatin	−47.30	5-Fluorouracyl	−63.48
5-Fluorouracyl	−34.46	Doxorubicin	−50.00
Irinotecan	−7.50	Cisplatin	−29.37
Epirubicin	−3.45	Docetaxel	−13.64
Cisplatin	8.36	Epirubicin	−6.90
Olaparib	11.86	Methotrexate	−2.56
Methotrexate	12.82	Irinotecan	−2.21
Docetaxel	27.27	Tamoxifen	30.66
Tamoxifen	34.12	Olaparib	36.86

## Data Availability

Data are contained within the article.

## Supplementary Materials

The following supporting information can be downloaded at https://www.mdpi.com/article/10.3390/cimb46110740/s1.
