# Supplementary material for: Complementary Treatment of Breast Cancer Cells with Different Metastatic Potential with Iscador Qu in the Presence of Clinically Approved Anticancer Drugs
Source: Curr Issues Mol Biol. 2024 Nov 5;46(11):12457–80. doi: 10.3390/cimb46110740 (PMC11593002; doi:10.3390/cimb46110740)

Supplementary Materials

Complementary Treatment of Breast Cancer Cells with Different Metastatic Potential with Iscador Qu in the Presence of Clinically Approved Anticancer Drugs

Ivan Iliev [1], Iana Tsoneva [2], Aleksandrina Nesheva [2], Galya Staneva [2*], Bozhil Robev [3], Albena Momchilova [2] and Biliana Nikolova [2*]

[1] Institute of Experimental Morphology, Pathology and Anthropology with Museum, Bulgarian Academy of Sciences, Acad. G. Bonchev Str., Bl. 25, Sofia, 1113, Bulgaria; taparsky@abv.bg

[2] Institute of Biophysics and Biomedical Engineering, Bulgarian Academy of Sciences, Acad. G. Bonchev Str., Bl. 21, Sofia, 1113, Bulgaria; itsoneva@bio21.bas.bg (I.T.); nescheva@gmail.com (A.N.); g_staneva@yahoo.com (G.S.); albena_momchilova@abv.bg (A.M.); nikolova@bio21.bas.bg (B.N.)

[3] Department of Medical Oncology, University Hospital "Sv. Ivan Rilski", 15 Acad. Ivan Geshov Blvd, Sofia, 1431, Bulgaria; bostro@abv.bg

* Correspondence: nikolova@bio21.bas.bg ; g_staneva@yahoo.com

Contents:

1. Figure S1. Fluorescence-acitvated cell sorting (FACS) analysis of Annexin V-FITC apoptosis assay of cells…….. ……………………………………………………………p.2

2. Cell cycle analysis using Fluorescence-acitvated cell sorting (FACS) analysis after staining with propidium iodide (PI)……………………………. …….………………. p. 3

Figure S1. Fluorescence-acitvated cell sorting (FACS) analysis of Annexin V-FITC apoptosis assay of cells from two cancer cell lines. Apoptosis levels were assessed in control and treated cells with Iscador Qu, Docetaxel, Cisplatin, and their combinations for MDA cells (A). Apoptosis levels were assessed in control and treated cells with Iscador Qu, Oxaliplatin, Olaparib, and their combinations for MCF-7 cells (B).

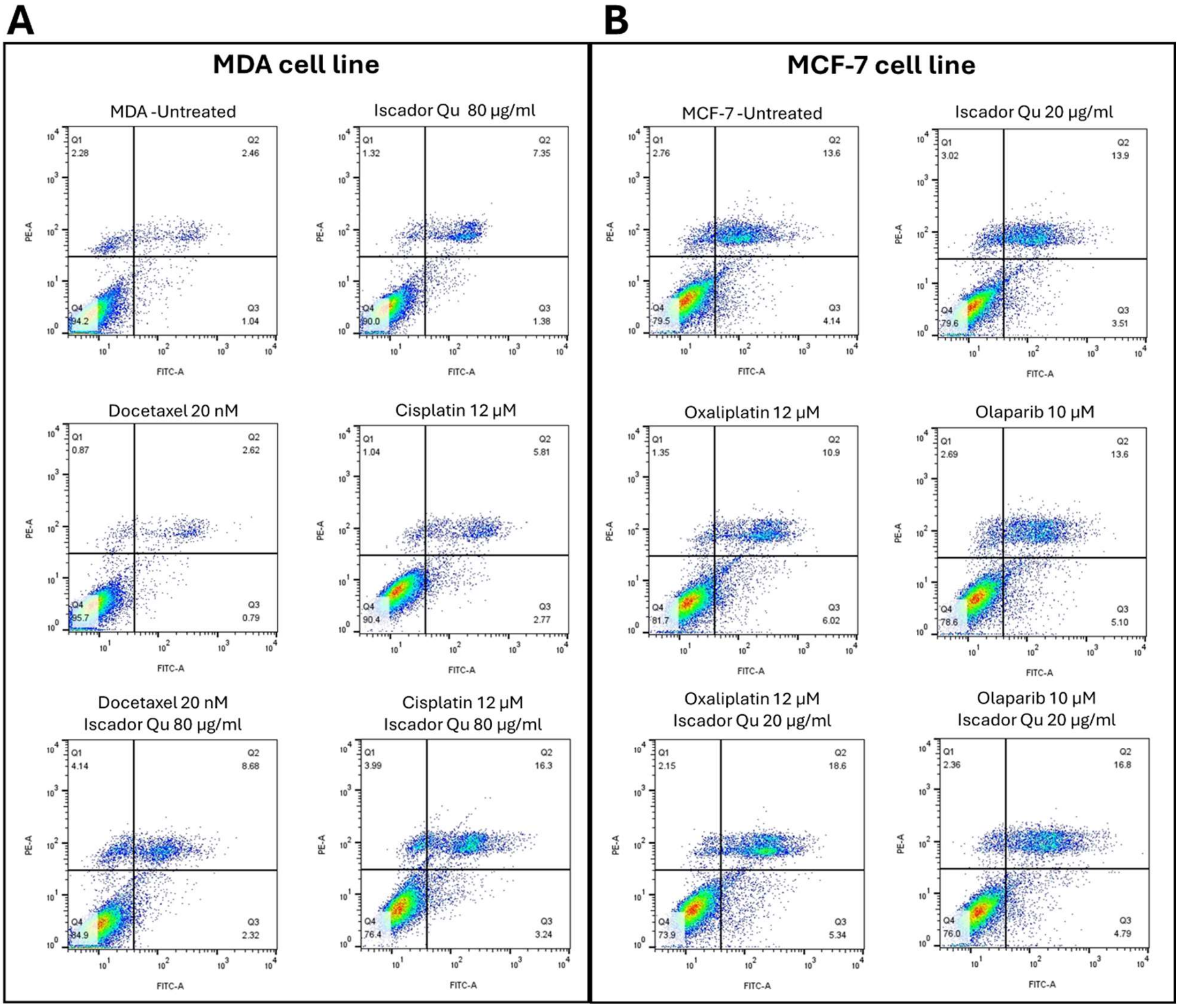

Figure S2. Cell cycle analysis using Fluorescence-acitvated cell sorting (FACS) analysis after staining with propidium iodide (PI). MDA cell cycle phase distributions for control cells and cells treated with Iscador Qu, Docetaxel, Cisplatin, and their combinations (A). MCF-7 line cell cycle phase distributions for control cells and cells treated with Iscador Qu, Oxaliplatin, Olaparib, and their combinations (B).

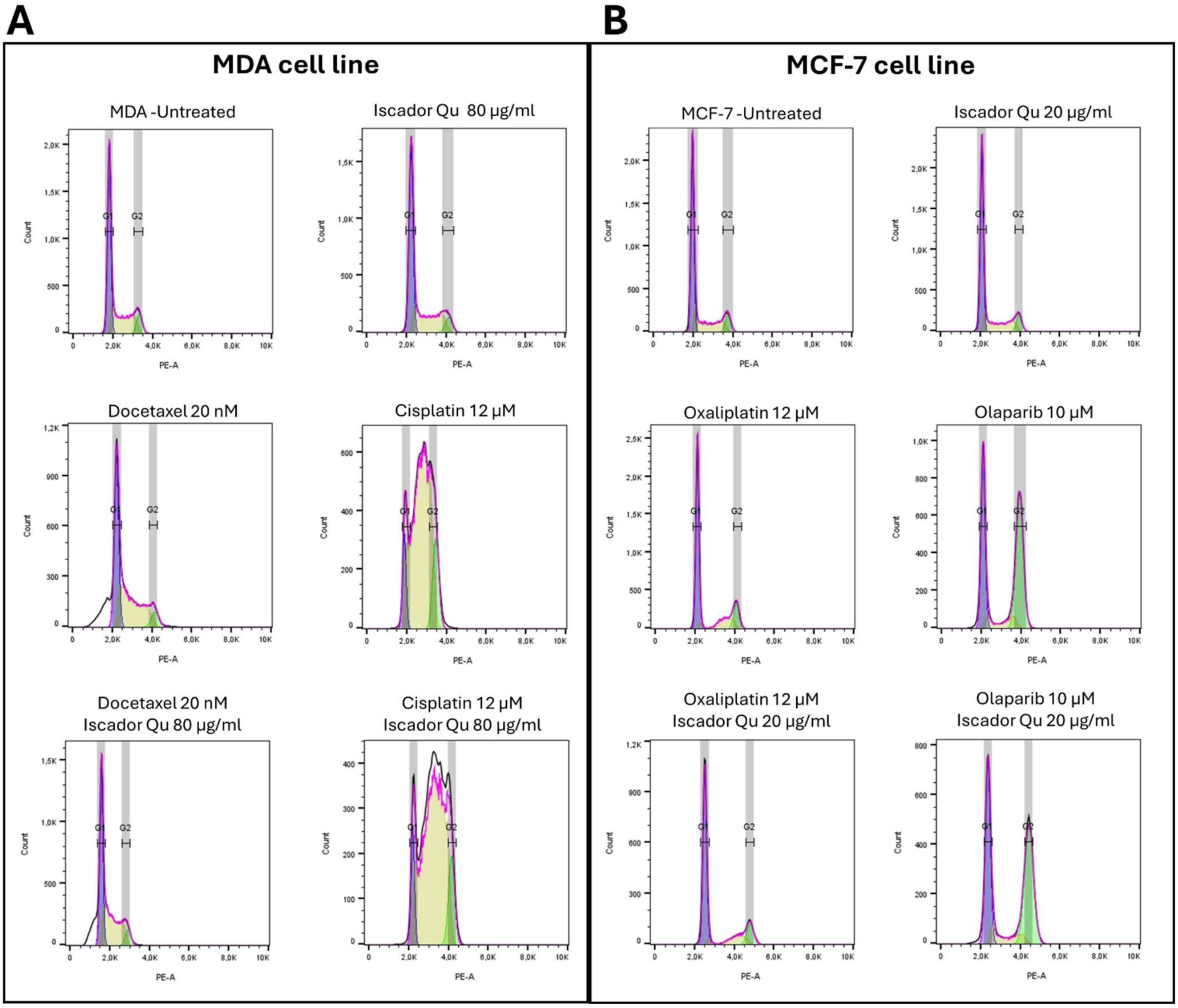

Supplement: Supplementary file 1 [file cimb-46-00740-s001.zip › cimb-3179061-supplementary.pdf]
